# Molecular Detection of Methicillin-Resistant *Staphylococcus aureus* by Non-Protein Coding RNA-Mediated Monoplex Polymerase Chain Reaction

**DOI:** 10.1371/journal.pone.0158736

**Published:** 2016-07-01

**Authors:** Cheryl Yeap Soo Yean, Kishanraj Selva Raju, Rathinam Xavier, Sreeramanan Subramaniam, Subash C. B. Gopinath, Suresh V. Chinni

**Affiliations:** 1 Department of Biotechnology, Faculty of Applied Sciences, AIMST University, Bedong, Malaysia; 2 School of Biological Sciences, Universiti Sains Malaysia, Penang, Malaysia; 3 Institute of Nano Electronic Engineering, Universiti Malaysia Perlis, Kangar, Perlis, Malaysia; 4 School of Bioprocess Engineering, Universiti Malaysia Perlis, Arau, Perlis, Malaysia; Naval Research Laboratory, UNITED STATES

## Abstract

Non-protein coding RNA (npcRNA) is a functional RNA molecule that is not translated into a protein. Bacterial npcRNAs are structurally diversified molecules, typically 50–200 nucleotides in length. They play a crucial physiological role in cellular networking, including stress responses, replication and bacterial virulence. In this study, by using an identified npcRNA gene (*Sau-02*) in Methicillin-resistant *Staphylococcus aureus* (MRSA), we identified the Gram-positive bacteria *S*. *aureus*. A *Sau-02-*mediated monoplex Polymerase Chain Reaction (PCR) assay was designed that displayed high sensitivity and specificity. Fourteen different bacteria and 18 *S*. *aureus* strains were tested, and the results showed that the *Sau-02* gene is specific to *S*. *aureus*. The detection limit was tested against genomic DNA from MRSA and was found to be ~10 genome copies. Further, the detection was extended to whole-cell MRSA detection, and we reached the detection limit with two bacteria. The monoplex PCR assay demonstrated in this study is a novel detection method that can replicate other npcRNA-mediated detection assays.

## Introduction

Over the last several years, there have been dramatic enhancements to *Staphylococcus aureus* strains to confer resistance against the antibiotic methicillin. The number of Methicillin-resistant *Staphylococcus aureus* (MRSA) infections is a worldwide concern, particularly in nosocomial settings, as MRSA accounts for 10 to 40% of the overall *S*. *aureus* isolates in the United States and European countries [1,2]. MRSA infection is serious and difficult to treat, and only a few antimicrobial agents are available for treating MRSA [3,4]. Therefore, it is important to generate a rapid and accurate detection of MRSA by using a novel molecule as the probe, which can yield higher sensitivity and specificity.

The classical methods of MRSA detection include biochemical tests, the agar dilution technique, and antibiotic susceptibility tests such as the Epsilometer test, Kirby-Bauer disc diffusion method, and immuno-diffusion technique. These techniques often provide ambiguous results and are time-consuming, usually requiring 5 to 7 days. Though, many molecular tests exist; PCR detection of MRSA are currently based on the *femA* gene [5–7], the *mecA* gene [6–9], or staphylococcal toxin genes such as *eta*, *etb*, *sea*, *seb*, *sec-1*, *sed*, *see*, and *tst* [10]. However, there are limitations when using toxin genes because they are present within the coding region [10,11] and are prone to mutation. Further, the *mecA* gene is used predominantly for detecting MRSA; it is conserved in MRSA but often yields unspecific result because it is specific not only to MRSA but also to other methicillin-resistant *Staphylococci* such as Methicillin-resistant *Staphylococcus epidermidis* [8]. The same issue occurs when using the *femA* gene for the molecular diagnosis of MRSA [12–14]. In contrast, detection by DNA hybridization has been found to be a sensitive method for identifying MRSA. However, DNA hybridization suffers from a few disadvantages, particularly in that more cells are required. Moreover, DNA extraction and immobilization on a membrane are tedious processes.

### Importance of npcRNA-mediated analysis

The importance of non-protein coding RNA (npcRNA) has been attested in past with functional evidence of its cellular milieu [15–17]. Further, there are evidences to support the use of npcRNA and other short nucleic acids in downstream analytical applications [16,18–20]. To generate a nucleic acid-mediated monoplex PCR, we selected an npcRNA gene as a tool for the detection of MRSA because npcRNA genes are more resistant to mutation than protein-coding genes. Point mutations tend to appear at the non-synonymous regions of genes that code for proteins [21], thus making the detection of a bacterium via PCR using a protein coding gene disadvantageous. In the case of an npcRNA mutation, the functional and secondary structure of the npcRNA would be altered [22]; hence, the bacterium may no longer survive, and detection would not be required. The current study presents a new approach to detecting MRSA by amplifying an npcRNA through PCR as a monoplex. The specificity and sensitivity of the monoplex PCR were studied. The results showed that the designed npcRNA primers are highly specific only to the selective bacteria with the *Sau-02* gene and are expressed in *Staphylococcus aureus* and MRSA.

Recent research has revealed that RNAs are key regulators in pathogens. Bacterial small npcRNAs are structurally diverse molecules that are 50–200 nucleotides long and belong to different classes [23]. The functions of npcRNA include the regulation of stress responses, plasmid and viral replication, bacterial virulence and quorum sensing. In general, npcRNA includes all RNAs except mRNA. Regulatory npcRNAs can base-pair to mRNAs that are acting in *trans-* or *cis-* [23] and can thereby either repress or activate translation efficiency by affecting the mRNA target stability [23] for genes that encode virulence proteins [24]. Further, some npcRNAs can bind and modulate the activity of proteins [25].

A recent study achieved the attomolar detection of multiple pathogens by using a npcRNA-mediated genosensor [26], and npcRNA proved to be a novel diagnostic marker for effectively discriminating *Salmonella* species [18]. Herein, we have improved on the detection of MRSA by developing npcRNA-mediated monoplex PCR using the *Sau-02* gene. A total of 142 npcRNAs were identified in *S*. *aureus* [27]. *Sau-02* is present only in *S*. *aureus* including MRSA, even other genus with Methicillin resistance are not possessing *Sau-02* gene and hence it is specific to *Staphylococcus aureus*. Thus it can facilitate downstream analyses such as molecular detection.

## Materials and Methods

### Bacterial strains

The bacterial isolates in this study used were acquired from Universiti Sains Malaysia (USM); and AIMST University, Malaysia. All bacterial isolates were maintained at -80°C in the recommended storage solution and were revived by inoculation into LB media at 37°C with shaking condition prior to monoplex PCR.

### Polymerase chain reaction (PCR)

The specific primers that recognize only *Staphylococcus aureus*/MRSA were detected based on the *Sau-02* gene. The forward and reverse primers were *Sau-02*-F: 5'- GTAAAAAGACGACATGCAGGAA-3' and *Sau-02*-R: 5'- CCATCATTTCAAAACTTTGACA -3'. The PCR master reagent mix contained 20 pmol of primers for the *Sau-02* gene, 1X PCR buffer, 2.5 mM MgCl_2_, 1 mM dNTPs, 1U DNA Polymerase and DNA template. PCR grade water was used as a negative control by replacing the DNA to evaluate the occurrence of contaminated DNA. The mixture was vortexed briefly. PCR amplifications were carried out using a Bio-Rad DNA Engine thermal cycler with single initial denaturation step (95°C for 300 s), 34 cycles of denaturation (95°C for 30 s), annealing (61°C for 30 s), and extension (72° for 30 s), followed by a final extension step (72°C for 600 s). The amplified products were resolved using agarose gel (1.5%) electrophoresis, followed by staining with ethidium bromide, and were visualized under appropriate UV illumination.

### Optimization by gradient PCR

Gradient PCR is one potential strategy to reduce non-specific annealing and amplification. In the present study, using the gradient function of the universal block, a gradient of 54°C to 62°C was set for the MRSA primers. By varying the annealing temperature in each row, we created 8 discrete annealing temperatures and found that 61°C was the optimal annealing temperature to minimize unspecific annealing of the primers. Thus, further analyses were carried out using constant annealing at 61°C.

### Determination of the analytical specificity of the *Sau-02* gene

Specificity test was performed using 18 *S*. *aureus* strains and 14 non-*Staphylococcus aureus* strains to determine the specificity of the *Sau-02* gene. The other steps were followed as mentioned above.

### Determination of the analytical sensitivity of the *Sau-02* gene

To determine the detection sensitivity of the *Sau-02* gene, genomic DNA or whole cells from the test strains were used. To test the detection limit of the npcRNA-mediated monoplex PCR using genomic DNA, different amounts of genomic DNA were extracted from cultured cells, and the templates were serially diluted from 350 ng to 35 ag per PCR. Similarly, the limit of detection of the monoplex PCR with whole bacterial cells was determined by using 2 μl of template from cultured broth of 10-fold serially diluted bacterial culture [10^-1^ to 10^−10^] suspensions ranging from TNTC (Too Numerous To Count) to 7 cells/100μl.

## Results and Discussion

### Analysis of the location of the *Sau-02* gene

**Fig 1:**
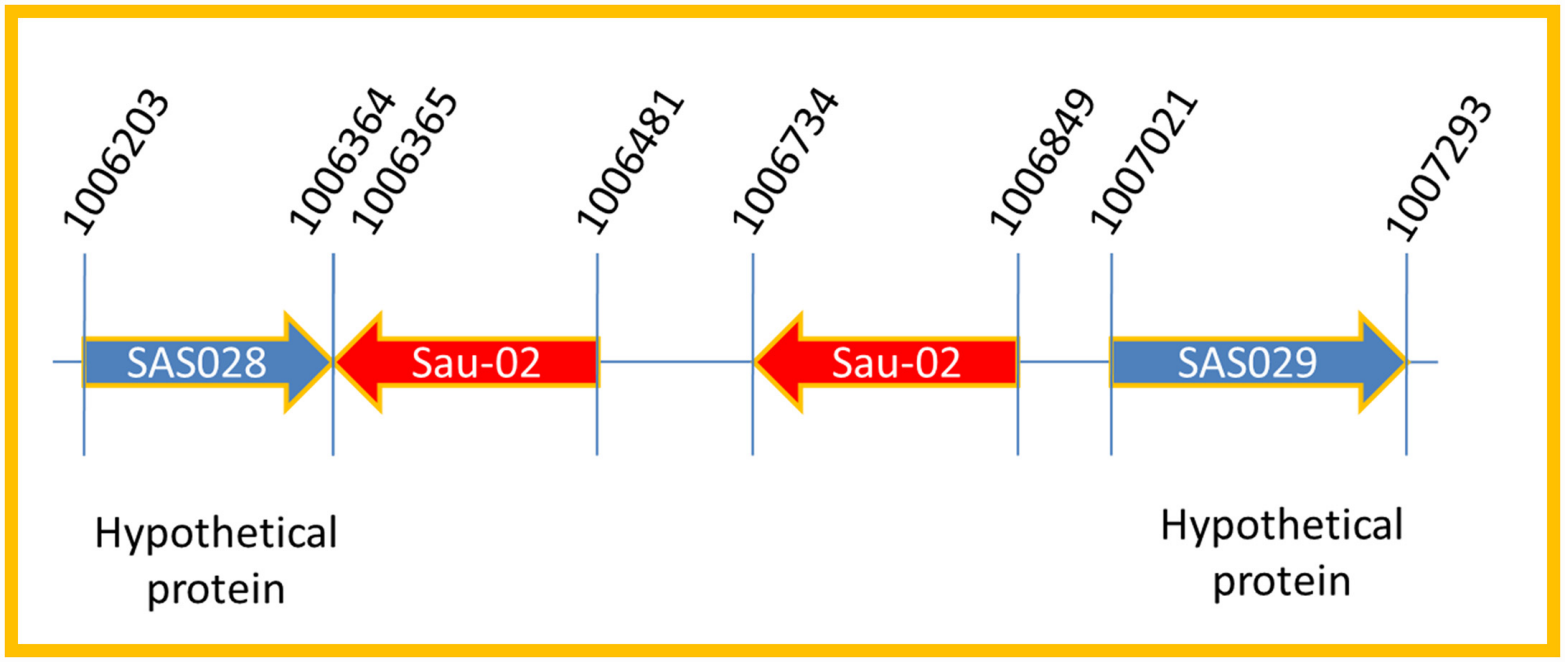
Location of preferred non-protein coding RNA. Location of the *Sau-02* gene is shown. Other genes responsible for hypothetical proteins are also shown.

*Staphylococcus aureus* is an opportunistic bacterium considered as a pathogen for significant infection to human and infects skin and respiratory system. Increasing researches on *S*. *aureus* in the past attested the importance of *S*. *aureus* strains with studies on metabolic pathways and analysis on genes [28,29]. *Sau-02* is one of the recently identified npcRNAs exclusively present in *S*. *aureus* determined through blastn search. Before being utilized for analysis by monoplex PCR, the location of the *Sau-02* gene was analyzed. Based on this preliminary analysis, it was found that the *Sau-02* gene is located in two regions of the *S*. *aureus* genome. These two copies are located in the regions of 1006365–1006481 and 1006734–1006849. Moreover, these *Sau-02* genes are located between two hypothetical protein genes: *SAS028* (1006203–1006364) and *SAS029* (1007021–1007293) in the opposite orientation (Fig 1).

### Determination of the *Sau-02* gene’s specificity using different bacterial species

**Fig 2:**
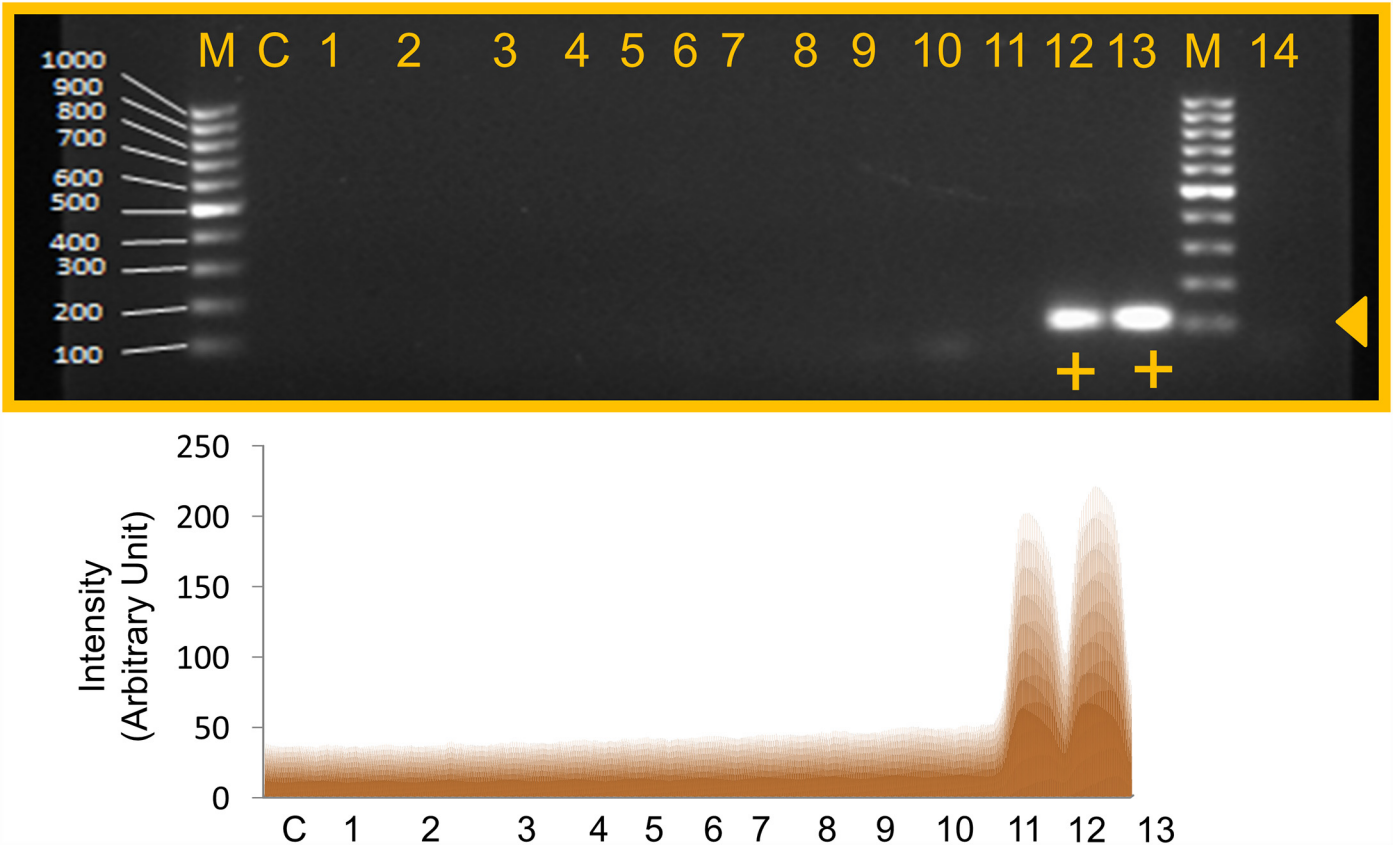
Specificity of MRSA PCR using various bacteria. M- 100bp DNA ladder; C- control; 1- *Bacillus subtilis;* 2- *Salmonella typhi;* 3- *Shigella flexneri;* 4- *Salmonella typhimurium;* 5- *Staphylococcus epidermidis;* 6- *Pseudomonas aeruginosa;* 7- *Aeromonas hydrophila;* 8- *Acinetobacter baumannii*.; 9- *Citrobacter freundii;* 10- *Enterobacter aerogenes;* 11- *Klebsiella pneumoniae;* 12- *Staphylococcus aureus*; 13- Methicillin-resistant *Staphylococcus aureus*; 14- *Vibrio cholerae*.’+’ indicates a positive result. Triangle arrowhead shows the band position. Lower panel is the scanned image from the ImageJ software.

The reliability of analytical technique developed is determined by its specificity and sensitivity. Specificity is a prime step for analytical development and can enhance the sensitivity of a given system [30]. In the current study, the specificity of the PCR assay was evaluated using 12 non-*Staphylococcus aureus* species and 2 *Staphylococcus aureus* strains. These bacterial species included *Bacillus subtilis*, *Salmonella typhi*, *Shigella flexneri*, *Salmonella typhimurium*, *Staphylococcus epidermidis*, *Pseudomonas aeruginosa*, *Aeromonas hydrophila*, *Acinetobacter baumannii*., *Citrobacter freundii*, *Enterobacter aerogenes*, *Klebsiella pneumoniae*, *Vibrio cholerae*, *Staphylococcus aureus* and MRSA. Based on monoplex PCR analysis using specific primers for the *Sau-02* gene, the PCR amplified products were resolved on agarose gel. From this analysis, it was obvious that the *Sau-02* gene was highly specific to both *S*. *aureus* and MRSA (lanes 12 and 13 in Fig 2). The other bacterial species showed no amplifications (lanes 1 to 11 and 14 in Fig 2). The scanning profile analysis was done using the ImageJ software [31], as shown in Fig 2 (lower panel).

### Determination of the *Sau-02* gene’s specificity using MRSA strains

**Fig 3:**
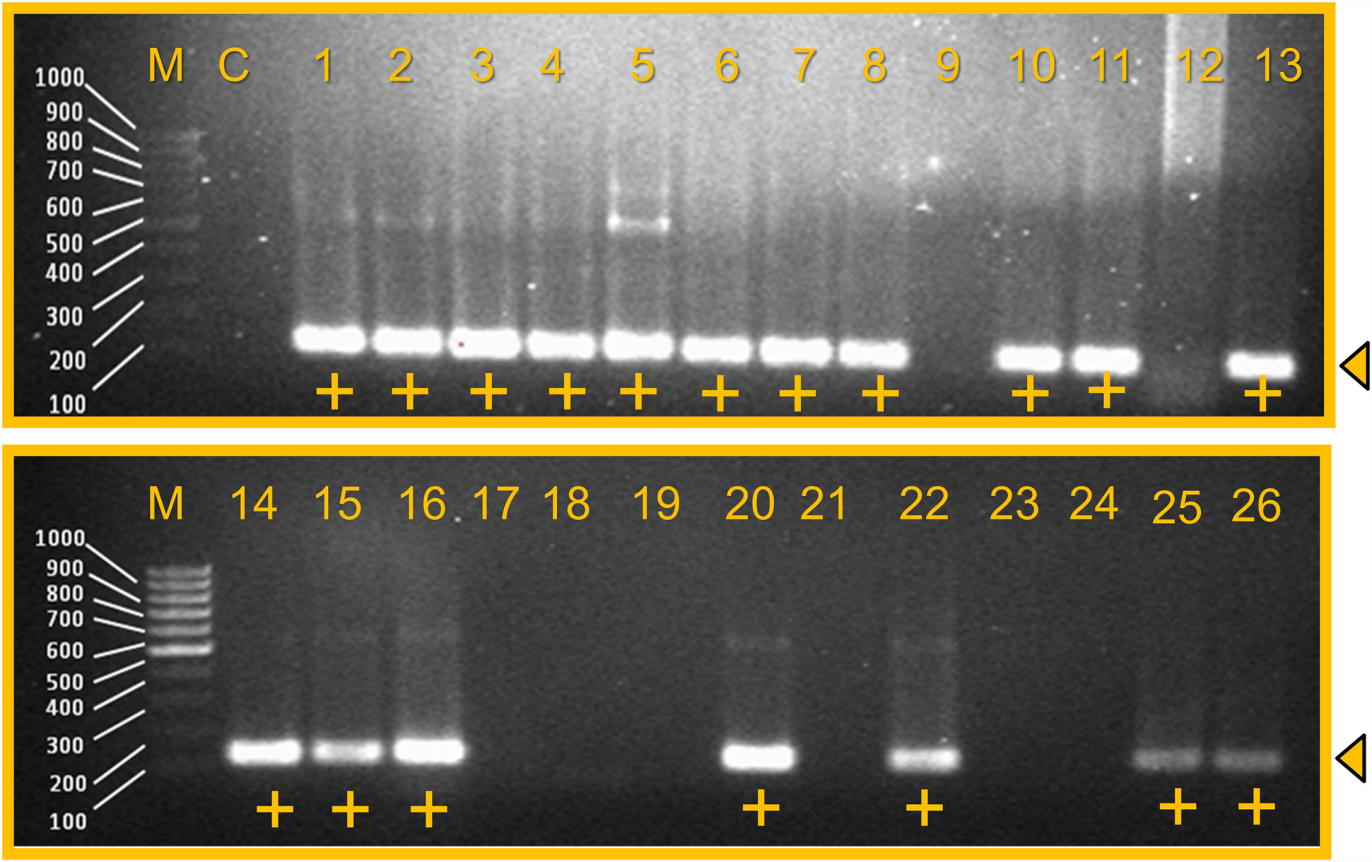
Specificity of MRSA PCR using 17 strains of MRSA. M- 100 bp DNA ladder; C- Negative control; 1- MRSA HA 1; 2- MRSA HA 2; 3- MRSA HA 3; 4- MRSA HA 4; 5- MRSA HA 5; 6- MRSA HA 7; 7- MRSA HA 9; 8- MRSA HA 10; 9- *Staphylococcus epidermidis*; 10- MRSA HA 12; 11- MRSA K 1; 12- *Bacillus subtilis*; 13- MRSA K 3; 14- MRSA K 5; 15- MRSA K 6; 16- MRSA S 2; 17- *B*. *thuringiensis*; 18- *Enterobacter aerogenes*; 19- *Acinetobacter baumannii*; 20- MRSA S 8; 21- *Staphylococcus haemolyticus*; 22- MRSA S 10; 23- *Salmonella typhi*; 24- *Salmonella typhimurium*; 25-*Staphylococcus aureus;* 26- MRSA S 12. ‘+’ indicates a positive result. Triangle arrowhead shows the band position.

Similar to the above analysis, we also performed monoplex PCR using 18 *Staphylococcus aureus* strains. An agarose gel displaying the results of the specific PCR using 8 non *S*. *aureus* species, 17 strains of MRSA and 1 *S*. *aureus* is shown in Fig 3. The analytical specificity testing was positively identified in all MRSA strains and *Staphylococcus aureus*, where amplicons of 110 bp were detected, thus indicating the occurrence of the target *Sau-02* gene in *Staphylococcus aureus* and MRSA only.

### Determination of the sensitivity of *Sau-02* detection using genomic DNA

**Fig 4:**
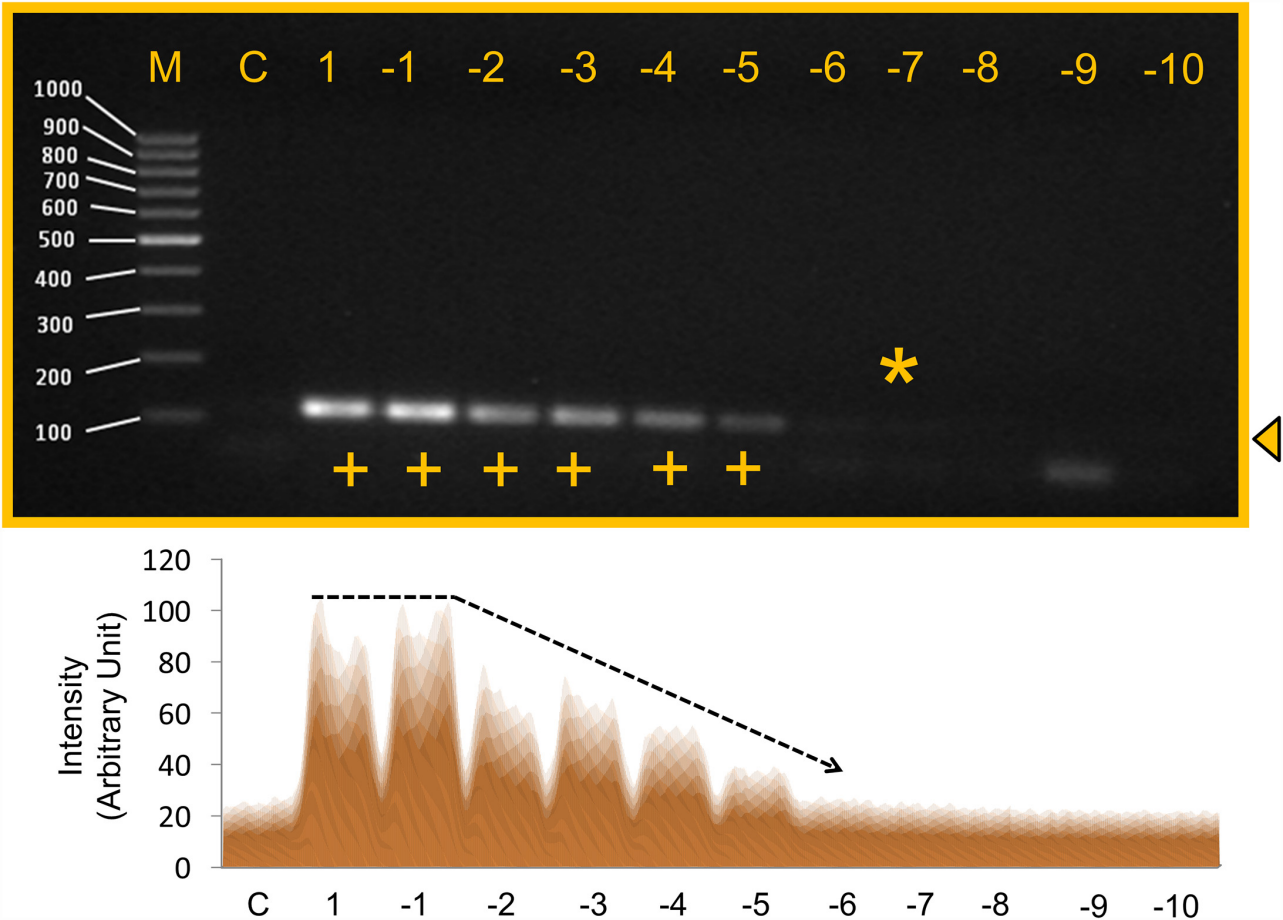
Detection limits of MRSA using genomic DNA. Lane M: Fermentas 100 bp DNA ladder; Lane C: Negative control (339.515 ng/μl); Lane1 to Lane 11 are sequential dilutions with dilution factors from 1 to 10^−10^ respectively; Lane 1: 1 (339.52 ng/μl); Lane 2: 10^−1^(33.95 ng/μl); Lane 3: 10^−2^ (3.39 ng /μl); Lane 4: 10^−3^ (339.52 pg /μl); Lane 5: 10^−4^(33.952 pg/μl): Lane 6: 10^−5^(3.39 pg /μl); Lane 7: 10^−6^ (339.52 fg /μl): Lane 8: 10^−7^ (33.95 fg/μl); Lane 9: 10^−8^ (3.39 fg /μl); Lane 10: 10^−9^(339.52 ag/μl); Lane 11: 10^−10^ (33.952 ag/μl). ‘+’ indicates a positive result. Star shows the sensitivity limit. Triangle arrowhead displays the band position. Lower panel is the scanned image from ImageJ software. The trend line for expression is drawn.

The analytical sensitivity (limits of detection, LOD) of the *Sau-02* gene was determined by testing serially diluted genomic DNA stock extracted from MRSA. The sensitivity test was performed to establish the MRSA detection limit. This test was conducted to check the minimal concentration of template that is required to detect MRSA. The genomic DNA was serially diluted to 10^−10^ from the stock, representing concentrations of 339.52 ng/μl to 33.952 ag/μl. From these results, it was noted that MRSA is detectable until the concentration of 10^−7^ dilution (33.95 fg/μl) calculated to be ~10 genomic copies of MRSA (Fig 4). The 50% of the highest intensity was observed with 339.52 pg/μl of genomic DNA (Fig 4).

### Determination of the sensitivity of *Sau-02* detection using whole bacterial cell

**Fig 5:**
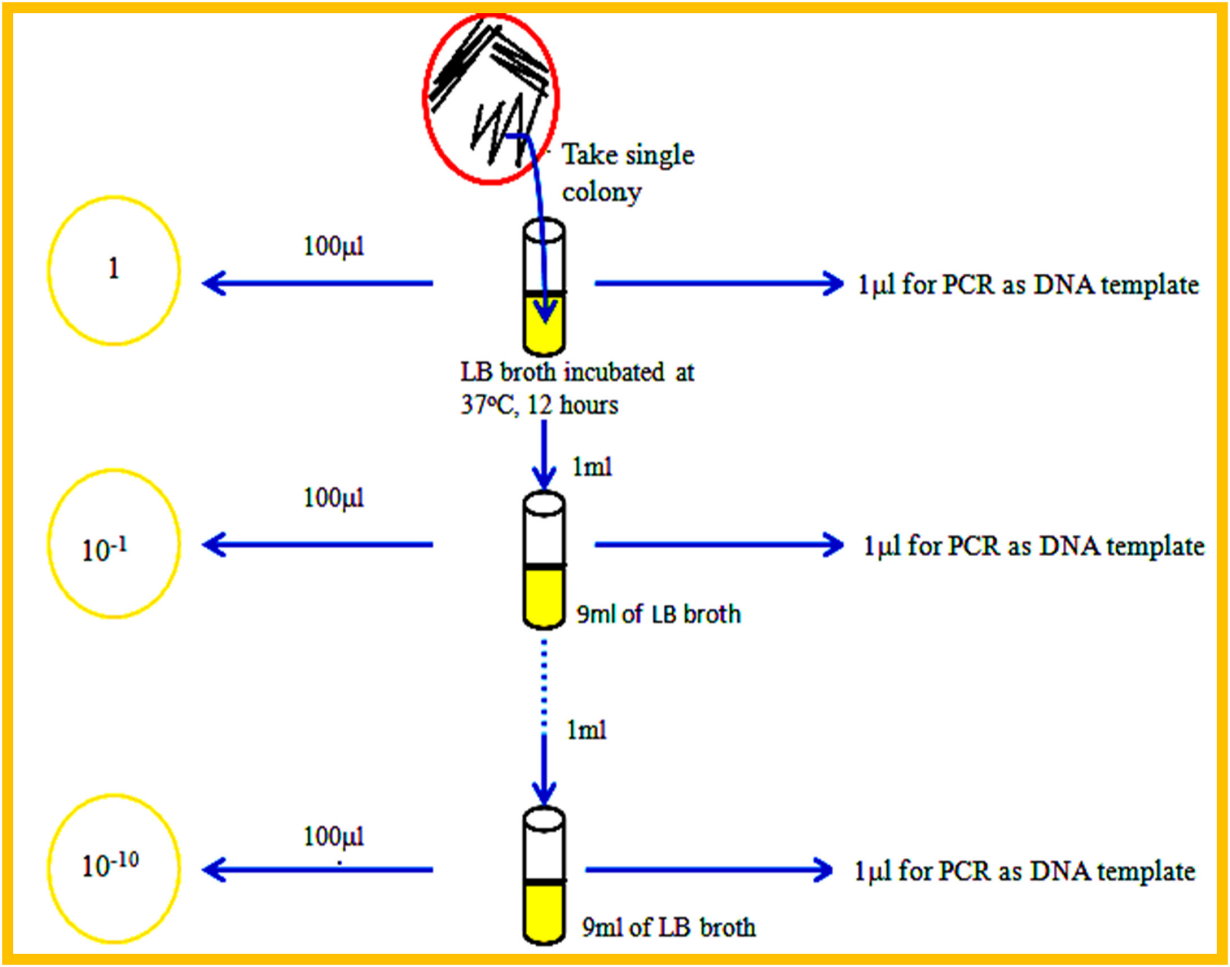
Schematic for the preparation of culture dilutions. Preparations of initial dilutions are shown. Other dilutions were prepared in similar way.

**Fig 6:**
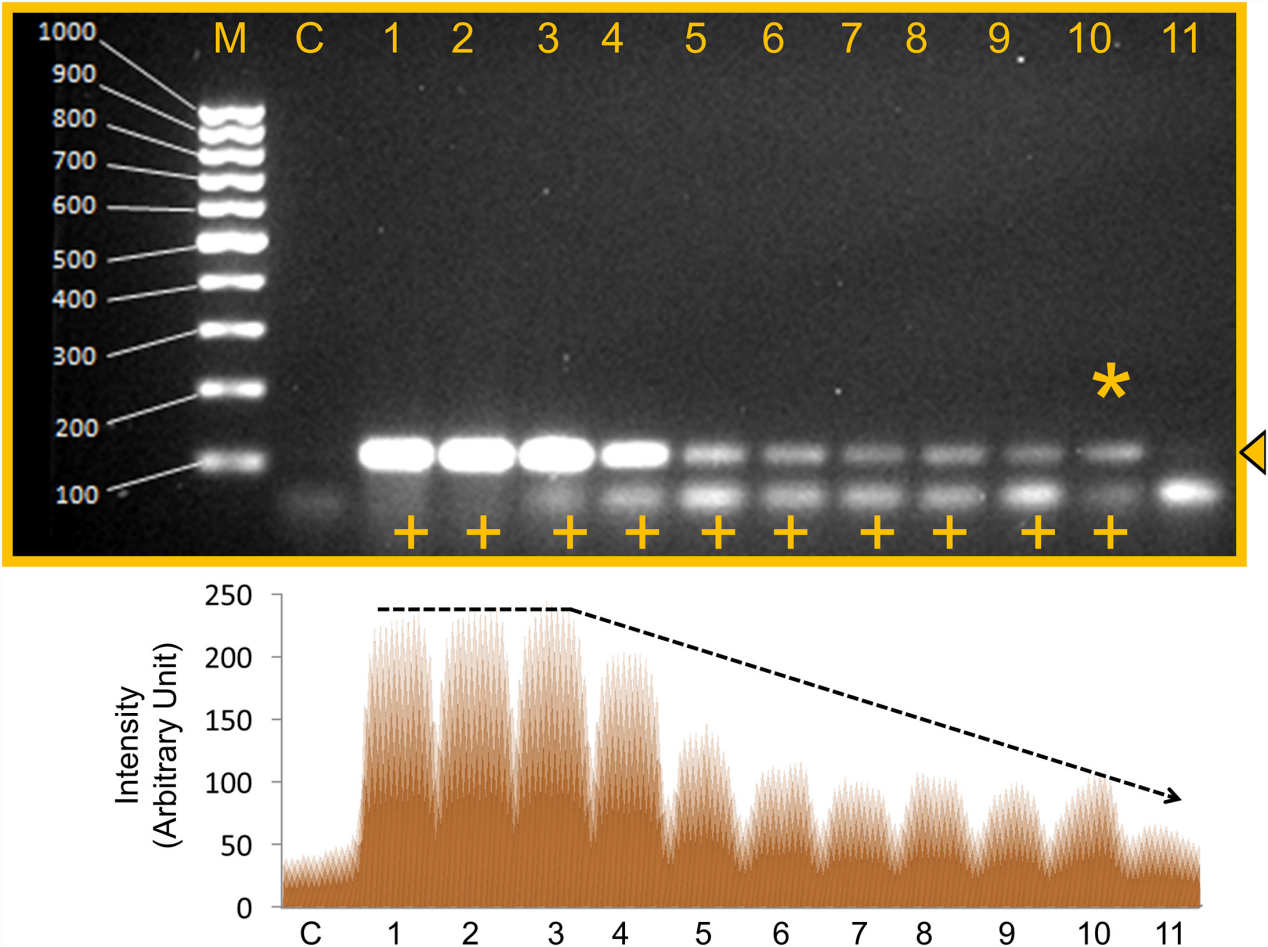
Detection limits of *S*. *aureus* using whole cell. Lane M: Fermentas 100 bp DNA ladder; Lane C: Negative control; Lane1 to Lane 11 are sequential dilutions with dilution factors from 1 to 10^−10^ respectively; Lane 1: 1; Lane 2: 10^−1^; Lane 3: 10^−2^; Lane 4: 10^−3^; Lane 5: 10^−4^; Lane 6: 10^−5^; Lane 7: 10^−6^; Lane 8: 10^−7^; Lane 9: 10^−8^; Lane 10: 10^−9^; Lane 11: 10^−10^. ‘+’ indicates a positive result. Star shows the sensitivity. Triangle arrowhead displays the band position. Lower panel is the scanned image from ImageJ software. The trend line for expression is drawn.

Whole cell detection has been an interesting and preferred strategy to detect an organism using the appropriate probe [32]. To give weight to the detection strategy shown here with monoplex PCR, we performed npcRNA-mediated whole cell detection. The bacterial culture was serially diluted as shown in the Fig 5. The numbers of colonies were estimated in all the dilutions per 100 μl of bacterial culture as shown in Table 1. From the results, it is clear that the current npcRNA-mediated monoplex PCR could detect bacterial dilutions up to 10^−9^ [~2 bacteria/reaction] (Fig 6). The 50% of the highest intensity was observed with 10^−8^ dilution having ~7 bacteria (Fig 6). The scanning profile obtained using ImageJ software has also shown a clear trend line for the amplification of the *Sau-02* gene from MRSA.

**Table 1:** Estimation of bacterial colonies from diluted samples.

Dilution factor	Number of bacterial colonies (x 10^2^)
1	TNTC
-1	TNTC
-2	TNTC
-3	TNTC
-4	TNTC
-5	TNTC
-6	TNTC
-7	TNTC
-8	705
-9	88
-10	7

TNTC—too numerous to count

*S*. *aureus* and MRSA infections are the worldwide challenge in healthcare. The current need in clinical microbiology is to develop an accurate detection method that is highly sensitive and specific. The World Health Organization recently structured an action plan against antimicrobial resistance which involves a series of approaches including a novel strategy for the diagnosis of pathogens [33]. A DNA microarray was developed representing genes coding house- keeping proteins, virulence factors and antibiotic determinants to detect bacteremia causing *S*. *aureus*, *E*. *coli*, *P*. *aeruginosa* [34]. Despite the accuracy of this method, it suffers from reduced sensitivity due to single or point mutation in the long probes used in microarrays. Application of PCR based on *16S* rRNA has replaced time consuming culture based detection until the availability of genome sequences [35]. Currently, detecting *mec-A* gene by PCR is the gold standard to identify methicillin resistant *S*. *aureus* [36]. This detection method is limiting because of the presence of *mec-A* gene in non-*Staphylococcus aureus* species [37] and non-*Staphylococcus* strains [38]. The two copies of *Sau-02* npcRNA gene present in *Staphylococcus aureus* and MRSA are highly specific and conserved. Hence, we developed the npcRNA based detection using *Sau-02* gene. Most of the detection studies carried out qualitatively to show the presence or absence of MRSA [36,39,40]. However, in our study we showed the detection level of genomic DNA equivalent to genome copies and whole cell MRSA as ~10 and 2 respectively. From the present investigation, we recommend that the *Sau-02* gene shall be exclusively used for the detection of *S*. *aureus*. Similarly, the *Sau-02* and *mecA* genes shall be used to detect MRSA. This approach will enhance the accuracy, sensitivity, specificity, speed, and cost effectiveness in the detection of *S*. *aureus* and MRSA.

## Conclusion

Non-protein coding RNA (npcRNA) is a widely accepted functional molecule (50–200 nucleotides long) that displays various vital functions. Herein, we used an npcRNA gene (*Sau-02*), to detect *S*. *aureus* and MRSA. A monoplex PCR assay was designed that demonstrated high sensitivity and specificity. The specificity of *Sau-02* gene was determined by testing 14 non-*S*. *aureus* bacterial species (10 Gram negative and 4 Gram positive) and 18 *S*. *aureus* including 17 MRSA strains. This study proved the detection of MRSA to the level of 34 fg genomic DNA equivalent to ~10 genomic copies of MRSA. The high sensitivity of the detection using whole cells of MRSA is experimentally shown to detect to the level of 10^−9^ dilution i.e., ~2 bacterial cells/reaction. The npcRNA-mediated monoplex PCR assay shown in this study demonstrates a novel and sensitive detection method that can be implemented in other systems.
